# Aortic Clamping Strategy During Coronary Artery Bypass Grafting: Partial or Single? A Propensity-Matched Analysis

**DOI:** 10.3390/jcdd13100502

**Published:** 2026-10-07

**Authors:** Osman Fehmi Beyazal, Zeki Temiztürk, Mustafa Karaarslan, Cemalettin Aydın, Mehmet Okan Donbaloğlu, Hasan Tezcan, Eylem Özköksal Kurtdişi, Nihan Kayalar, Mehmed Yanartaş

**Affiliations:** Department of Cardiovascular Surgery, Basaksehir Cam and Sakura City Hospital, Istanbul 34480, Turkey; zekitmztrk5806@gmail.com (Z.T.); karaarslanmustafa123@gmail.com (M.K.); cemalettinaydin1@hotmail.com (C.A.); donbalogluokan@hotmail.com (M.O.D.); hasan.tezcan44@hotmail.com (H.T.); eylemozkoksal97@hotmail.com (E.Ö.K.); nkayalar@hotmail.com (N.K.); myanartas@yahoo.com (M.Y.)

**Keywords:** partial aortic clamp, single aortic clamp, coronary artery bypass graft, atrial fibrillation, coronary artery disease

## Abstract

Background: This study aimed to investigate the relationship between partial aortic clamping (PAC) and single aortic clamping (SAC) techniques during isolated coronary artery bypass grafting (CABG) and their impact on clinical outcomes. Methods: Between 2022 and 2026, 974 patients who underwent isolated CABG were retrospectively evaluated. The study cohort was initially divided based on the operative technique into Group A (PAC, n = 626) and Group B (SAC, n = 348). To account for baseline clinical disparities, a 1:1 propensity score-matched (PSM) analysis was performed, yielding two balanced subgroups: Group C (PAC, n = 218) and Group D (SAC, n = 218). Results: Following propensity matching, the median cross-clamp (XCL) time was significantly lower in the PAC group compared to the SAC group (73 min vs. 100 min, *p* < 0.001). No statistically significant differences were observed between the balanced groups regarding postoperative exploration, cerebrovascular accidents, continuous renal replacement therapy requirements, or all-cause in-hospital mortality. The incidence of NOAF was significantly lower in the PAC group than in the SAC group [24 (11.0%) vs. 49 (22.5%), *p* = 0.001; absolute risk difference 11.5%, 95% CI 4.5–18.4%; number needed to treat ≈9]. Multivariate logistic regression analysis identified the SAC technique as an independent predictor of NOAF (*p* = 0.001, OR= 2.531 [95% CI, 1.442–4.441]). Conclusions: This study demonstrated no significant differences between the PAC and SAC techniques regarding postoperative bleeding, blood product utilization, and major neurological or renal complications. However, the PAC strategy was associated with a significantly lower incidence of NOAF compared to the SAC technique.

## 1. Introduction

Coronary artery bypass grafting (CABG) remains the most common surgical intervention in adult cardiac surgery, with approximately 200,000 procedures performed annually in the United States alone [1]. There are two commonly used strategies in on-pump CABG: single aortic clamping (SAC) and partial aortic clamping (PAC). Both techniques are frequently used depending on the patient’s vascular structure, the type of operation, and the preference of the surgeon. However, there is no definitive data on which technique is superior to the other. Although many studies have been conducted on this subject, there are still conflicting results. Chen et al. found no difference in postoperative stroke and 30-day mortality in their study comparing the SAC and PAC strategies [2]. Daniel et al. reported that the risk of postoperative stroke decreased with the SAC technique [3]. Hammon et al. also reported that surgery with SAC and minimal aortic manipulation may be equal to or superior to off-pump surgery in terms of neurophysiological deficits [4].

Most of the studies have compared postoperative stroke, neurological deficit, or basic postoperative outcomes. However, due to the difference in cross-clamp (XCL) times between the groups, not only cerebrovascular accident (CVA) but also complications related to XCL may differ between the groups. Consequently, with this investigation, we aimed to conduct a rigorous, propensity score-matched evaluation comparing the clinical impacts of PAC versus SAC techniques in a high-volume isolated CABG cohort.

## 2. Methods

This study was designed as a retrospective, single-center study evaluating a total of 1095 patients. All patients over the age of 18 who underwent isolated CABG at Istanbul Basaksehir Cam and Sakura City Hospital between 28 October 2022 and 22 May 2026 were included. Beating-heart procedures (n = 84), minimally invasive procedures (n = 22), and patients without proximal anastomosis surgery (n = 15) were excluded from the study. Following the exclusion criteria, 974 patients were included in the study. The patients were divided into two groups. Group A (n = 626): PAC group; Group B (n = 348): SAC group. Groups were matched, and new subgroups were obtained using propensity score matching (PSM), which included parameters such as age, cardiopulmonary bypass (CPB) times, the number of bypass grafts, diabetes mellitus (DM), and hypertension (HT). Group C (n = 218): PAC group; Group D (n = 218): SAC group. The flowchart for the study is presented in Figure 1.

Regarding the aortic clamping strategy, assessment of aortic plaque was performed in all patients via preoperative transthoracic echocardiography (TTE) and intraoperative manual palpation, and in a few select patients via thoracic computed tomography (CT), without the use of quantitative criteria. Thoracic CT or intraoperative echocardiography was not routinely performed for all CABG patients. The clamping strategy was decided according to the status of the vascular structure, type of operation, additional cardiac procedures, number and locations of vessels to be anastomosed, and most commonly the preference of the surgeon. In our clinic, continuous electrocardiogram (ECG) monitoring is performed only in the intensive care unit (ICU). During ward care, monitoring is conducted solely via 12-lead ECG until the patient is discharged. An episode of atrial fibrillation detected and documented by 12-lead ECG for the first time in a patient with no prior clinical history of atrial fibrillation was considered new-onset atrial fibrillation (NOAF). CVA was defined as a neurological deficit that occurred during the entire follow-up period, confirmed by imaging methods, and validated by a neurologist. The mortality rate was defined as in-hospital mortality.

Basic demographic characteristics, medical history, preoperative and postoperative 1st-week TTE findings, preoperative, laboratory parameters, the European System for Cardiac Operative Risk Evaluation (EuroSCORE II), preoperative, during XCL and 1st-day postoperative arterial blood gas (ABG) lactate values, surgical procedure details, near-infrared spectroscopy (NIRS) findings before XCL and during XCL, inotrope doses, vasoactive inotrope score (VIS), amount of bleeding, blood product usage, postoperative complications, intubation time, hospital and intensive care unit (ICU) stay, and mortality were recorded by reviewing the medical records of all patients. Afterward, comparisons were made between these groups in terms of all these parameters.

This study was approved by Istanbul Basaksehir Cam and Sakura City Hospital (no: 2026-384, 16 July 2026). The study was conducted in accordance with the Declaration of Helsinki. The reporting of this study conforms to the STROBE guidelines (Supplementary Materials) [5].

## 3. Statistics

Data analysis was performed using IBM SPSS Statistics for Windows (version 27.0; IBM Corp., Armonk, NY, USA). Continuous parameters are expressed as medians with interquartile ranges and full minimum–maximum boundaries based on the Kolmogorov–Smirnov normality testing results. Categorical metrics are expressed as absolute frequencies and percentages. Intergroup comparisons of continuous, non-normally distributed parameters were performed using the Mann–Whitney U test, while categorical parameters were analyzed via Pearson’s chi-square or Fisher’s exact tests. To adjust for significant baseline confounding and treatment assignment bias between the PAC and SAC groups, a 1:1 nearest-neighbor PSM protocol was carried out without replacement, utilizing a caliper width of 0.1 SD. Covariates incorporated into the matching algorithm included age, CPB times, the total number of distal anastomoses, DM, and HT. The ultimate matching quality was verified using the standardized mean difference (SMD). For the comparison of NOAF between the matched groups, the absolute risk difference with its 95% confidence interval (CI), the relative risk, and the number needed to treat were also calculated. Independent predictors of NOAF were identified using multivariable logistic regression analysis in the matched cohort. Multicollinearity was assessed using variance inflation factors (VIF), calibration using the Hosmer–Lemeshow test, and discrimination using the area under the receiver operating characteristic curve (AUC). Statistical significance was defined as a two-sided *p*-value of less than 0.05. There were no missing observations for any of the primary clinical endpoints, nor for the covariates incorporated into the propensity score matching and multivariable logistic regression models; therefore, a complete-case analysis was performed for these evaluations.

## 4. Results

The complete baseline clinical profile, demographic parameters, chronic comorbidities, and operative details of the unmatched and matched cohorts are detailed in Table 1. The overall unadjusted study sample displayed a mean age of 60.3 ± 9.1 years, with 787 male patients (80.8%). In the initial comparison between Group A and Group B, no significant disparities were found regarding sex, height, or weight distribution. However, Group B was significantly older than Group A (median 62 vs. 60 years, *p* = 0.007). Baseline chronic comorbidities, preoperative ejection fraction (EF), and preoperative tricuspid annular plane systolic excursion (TAPSE) levels were comparable between the unmatched groups. Intraoperatively, the SAC group had a significantly higher number of distal grafts, accompanied by prolonged median XCL times (101 vs. 67 min, *p* < 0.001) and longer CPB times (136 vs. 122 min, *p* < 0.001) compared to the PAC group. Pre-clamping and post-clamping NIRS values did not diverge significantly. Following the PSM algorithm to balance these critical operative variables, covariate balance improved. Within the balanced subgroups (Group C and Group D), all baseline demographics and comorbidities were similar. Operative parameters, including CPB times, graft counts, and dual antiplatelet therapy status, were statistically comparable. The median XCL times remained significantly longer in the SAC group than in the PAC cohort (100 min vs. 73 min, *p* < 0.001).

A comparison of perioperative blood product utilization and mediastinal drainage for the matched cohorts is detailed in Table 2. The intraoperative administration of red blood cells (RBCs), fresh frozen plasma (FFP), and apheresis platelet suspensions (APS) was statistically equivalent between the arms. In the postoperative phase, while RBCs and APS usage showed no intergroup variation, the requirements for FFP were significantly higher in the SAC cohort than in the PAC cohort. No significant difference was found between the two groups in terms of postoperative bleeding amounts (median 725 mL vs. 700 mL, respectively, *p* = 0.09).

Detailed evaluations of perioperative laboratory measurements and ABG findings are documented in Table 3. Preoperative leukocyte counts, hematocrit, platelets, urea, creatinine, electrolytes, liver transaminases, CRP, HbA1c, Troponin T, and CK-MB markers were comparable. Furthermore, laboratory monitoring on the first postoperative day and first month demonstrated no statistical divergence between the groups. ABG evaluations, including preoperative pH and lactate levels, hematocrit, potassium, and lactate profiles during XCL, as well as first postoperative day pH and lactate levels, remained similar across both cohorts.

Early postoperative morbidity and mortality endpoints for the matched groups are summarized in Table 4. No significant differences were observed between the strategies regarding postoperative exploration, CVA, deep sternal wound infection, gastrointestinal bleeding, subsequent percutaneous coronary intervention (PCI), or the need for continuous renal replacement therapy (CRRT), intra-aortic balloon pump, or extracorporeal membrane oxygenation support. Similarly, intubation times, VIS, ICU stays, and total hospital stays were equivalent. In-hospital mortality rates were also statistically similar between the groups [5 (2.3%) in PAC vs. 7 (3.2%) in SAC, *p* = 0.55]. However, the incidence of NOAF was significantly lower in the PAC group compared to the SAC group [24 (11.0%) vs. 49 (22.5%), *p* = 0.001] (Figure 2). This corresponded to an absolute risk difference of 11.5% (95% CI, 4.5–18.4%) and a relative risk of 2.04, with a number needed to treat of approximately 9 (95% CI, 5–22).

The results of the multivariate logistic regression analysis evaluating independent predictors of NOAF are outlined in Table 5. No significant relationship was found between hypertension, diabetes mellitus, sex, chronic renal failure, emergency surgery, CPB time, chronic obstructive pulmonary disease, the number of bypass grafts, and EF and NOAF. A significant relationship was found between SAC technique and NOAF (*p* = 0.001, OR= 2.531 [95% CI, 1.442–4.441]). Similarly, a significant relationship was found between age and NOAF (*p* < 0.001, OR= 1.092 [95% CI, 1.055–1.130]). No significant multicollinearity was detected (all VIF values <1.6). The model showed adequate calibration (Hosmer–Lemeshow χ^2^ = 4.86, *p* = 0.77) and acceptable discrimination (AUC = 0.745; 95% CI, 0.676–0.813).

## 5. Discussion

The main complications associated with CABG are mortality, CVA, wound infection, prolonged ventilation, acute kidney injury (AKI), reoperation, and gastrointestinal bleeding. The reported complication rates after CABG are approximately 30.0% for postoperative atrial fibrillation, 12.3% for prolonged ventilation, 4.5% for renal failure, 3.5% for reoperation, 1.9% for CVA, and 0.4% for sternal wound infection [6]. There are many risk factors for these complications that are patient-specific and surgical technique-specific. Aortic clamping strategies are one of these risk factors. Although surgeries performed with facilitating devices for proximal anastomosis (Heartstring™, Maquet, CA) and PAS-Port (Cardica, Inc, California) have been reported, many centers still perform surgeries with aortic clamping [7]. Although there are studies comparing these techniques, a definitive consensus has not yet been reached. In this retrospective detailed study, we observed that the PAC technique was associated with less NOAF compared to the SAC technique.

The two most commonly used methods for aortic clamp strategy are PAC and SAC. In the SAC technique, all anastomoses are performed during XCL. In the PAC technique, proximal anastomoses are performed by removing the XCL and re-clamping the aorta. Since all anastomoses are performed with a single clamp in SAC, the XCL time is expected to be higher than in PAC, and this may pose a risk in terms of complications related to prolonged XCL time. In contrast, proximal anastomosis is frequently performed after each distal anastomosis in the SAC technique. With cardioplegic solutions administered at frequent intervals, they can be delivered to the myocardial tissue from both the native coronary arteries and the grafts. Although the expected XCL time is shorter in the PAC technique, this effect of cardioplegia cannot be utilized unless cardioplegia is given from the grafts. The cardioplegia to be administered will only reach the myocardium from the narrowed native coronary arteries. This disadvantage can be reduced by connecting additional lines to the venous grafts during cardioplegia administration. Apart from myocardial protection, re-clamping the aorta may pose a risk for CVA. For this purpose, many studies have been conducted comparing these techniques [2,3,4,8,9]. Some studies have also investigated the effects of these techniques on myocardial protection [10]. However, there is still no accepted gold standard method in this regard. CPB and XCL times, which are among the most important causes of complications after CABG, may differ between groups. This may also affect outcomes following CABG. In most of the studies conducted so far, these techniques have been compared frequently in terms of CVAs. In this study, complications after CABG were compared in detail between groups, and we investigated which technique was more beneficial.

After a strict selection criterion, we obtained a patient group with similar preoperative cardiac characteristics. Initial analysis of Groups A and B revealed differences in certain demographic data, such as age, and particularly in important operative characteristics like CPB times. As expected, due to the proximal anastomosis technique, XCL time was significantly lower in the PAC group. However, in the initial comparison, CPB times were also significantly higher in the SAC group. In the PAC technique, the heart is activated after the XCL is removed, and the warming phase begins earlier. The heart, which is warmed and activated earlier, may be ready to be weaned from CPB immediately after the proximal anastomoses are completed. However, in the SAC technique, the heart may need more time to be weaned from CPB after the XCL is removed. This could be one reason for the CPB time difference. However, Chu et al. found a 5% shorter CPB time but a 10% longer myocardial ischemia time in the SAC group, despite a similar number of distal anastomoses [8]. However, since CPB times can have a very significant impact on outcomes, PSM analysis was performed to match groups, and new subgroups with similar characteristics were created. Of course, XCL times were not included in this analysis. The main aim of the study was to investigate the effect of the expected difference in XCL times between aortic clamping techniques on postoperative outcomes.

In the SAC technique, metabolic demand can be reduced by administering cardioplegia from both native coronary arteries and grafts, and frequent flushing of lactic acid can be achieved [10]. However, in our study, there was no difference between the groups in terms of lactate values before surgery, during XCL, and on the first postoperative day. Postoperative EF and TAPSE values were similar between the groups. In addition, there was no difference in terms of postoperative VIS. Although XCL time was longer in the SAC group, these results can be considered indicators of adequate myocardial protection. Although shortening the XCL time is important for improving postoperative outcomes, it is also important to provide adequate myocardial protection through grafts after XCL. In fact, in our study, we observed similar postoperative outcomes between the groups except for NOAF.

In the PAC technique, re-clamping the aorta may increase the risk of aortic injury and dissection. Intraoperative dissection was not detected in our study. Intraoperative and postoperative blood product usage was also similar between groups. There was no difference between the groups in terms of postoperative amounts of bleeding. In addition, CPB has negative effects on the renal system, hepatic system, and hematological system in the postoperative period [11]. There were no significant differences in laboratory parameters between the groups. There was also no difference in terms of postoperative CRRT need between the groups. There was no significant difference between these two clamping techniques that would cause a substantial change in clinical and laboratory values related to the kidney and liver systems.

We did not observe any difference between the groups in terms of many cardiac and noncardiac complications. Only the NOAF frequency was lower in the PAC group. According to the results of multivariate regression analysis, we found a significant relationship between the SAC technique and NOAF (OR =2.531). Although many comparisons have been made in terms of NOAF risk with XCL time and cardioplegia types, there is not enough data in terms of the aortic clamping technique and NOAF risk. Eris et al. found postoperative AF in 10% of cases with the SAC technique and 15% with the PAC technique, but found no statistically significant difference [10]. The most important reason for this result may be directly related to the long XCL times in the SAC technique. Indeed, prolonged XCL time is associated with atrial fibrillation following CABG [12]. Our hypothesis is that the SAC technique does not directly cause NOAF, but may indirectly lead to a prolongation of XCL time, which in turn could be associated with the risk of NOAF. Additionally, the increased need for cardioplegia during this time may also have contributed to this. This suggests that methods aimed at shortening the XCL time can be beneficial in reducing NOAF and the associated risks. However, it would still be beneficial to investigate this issue with future randomized controlled trials.

CVA has been the most studied parameter in the studies to date to compare these two techniques. Many risk factors have been identified, including aortic manipulation, cannulation and decannulation stages, microthrombus embolism, hemodynamic changes, and prolonged hypoperfusion during CPB, atherosclerosis, female gender, advanced age, history of CVA, preoperative AF, carotid stenosis, and DM [3,13]. Although it is thought that the risk of CVA may be lower in off-pump surgery, in the randomized controlled trial of Diegeler et al., no significant difference was found between on-pump and off-pump CABG in terms of death and CVA in patients aged 75 years and older [14]. In comparison with the aortic clamp technique in on-pump surgery, studies show that the SAC technique reduces the risk of CVA [3,9,15], while there are also studies showing no difference between the techniques [2,8,16,17,18,19,20]. However, in our study, there was no significant difference in CVA between these two techniques. It would be beneficial to investigate this issue with randomized controlled studies in the future.

In our study cohort, there were no differences in intubation times, ICU stay, and hospital stays between the PAC and SAC groups. Only the ICU stay was slightly longer in the SAC group, but statistically, they were similar (*p* = 0.08). A significant reason for this could be NOAF. It was an expected result that patients with a higher NOAF rate would have a longer ICU stay. However, considering that the majority of patients who develop NOAF after CABG revert to normal sinus rhythm within a short period [21], the lack of difference regarding hospital stay and other complications may not be surprising. In absolute terms, the lower incidence of NOAF in the PAC group corresponded to approximately one case of NOAF fewer for every nine patients. However, given the retrospective observational design, this estimate reflects an association rather than a proven treatment effect. Chu et al. reported 1.9% and 1.8% mortality rates in the PAC and SAC groups, respectively, in the first 30 days [8]. We observed similar mortality rates between the PAC and SAC groups. Chen et al. also found no difference in 30-day mortality between the PAC and SAC groups [2]. These results are consistent with the literature. However, current findings support that neither technique is superior to the other in terms of mortality rates and length of hospital stay.

## 6. Limitations

This study is a retrospective and single-center study. This study is subject to selection bias and confounding by indication, as the clamping strategy was not randomized and may have been influenced by factors such as aortic calcification or surgical complexity. Aortic plaque grading could not be performed in sufficient detail to allow for assessment using a quantitative measure. We were unable to make a comparison regarding the types and compositions of cardioplegia. Both the type of cardioplegia and the antegrade or retrograde route of administration are important factors, especially for myocardial protection, and may affect postoperative outcomes. In addition, some patients in the PAC group received cardioplegia through additional lines connected to venous grafts during XCL, but this could not be compared. Comparisons could not be made due to insufficient data regarding graft configuration and coronary anatomy. Although confounding parameters such as left atrial diameter, electrolyte disturbances, and preoperative medications (such as beta-blockers and angiotensin-converting enzyme inhibitors) are associated with NOAF, they could not be compared between the groups. A more detailed comparison of the aortic clamping technique and ICU stay would be useful. Since the diagnosis of NOAF was established using a 12-lead ECG, particularly during ward monitoring, some cases of transient atrial fibrillation may have been overlooked. Finally, there is no medium- or long-term follow-up after discharge.

## 7. Conclusions

In conclusion, this propensity score-matched study demonstrated no significant differences between the PAC and SAC techniques regarding postoperative bleeding, blood product utilization, and major neurological or renal complications. However, the PAC strategy was associated with a significantly lower incidence of NOAF compared to the SAC technique. However, these findings need to be investigated in future randomized controlled trials.

## Figures and Tables

**Figure 1:**
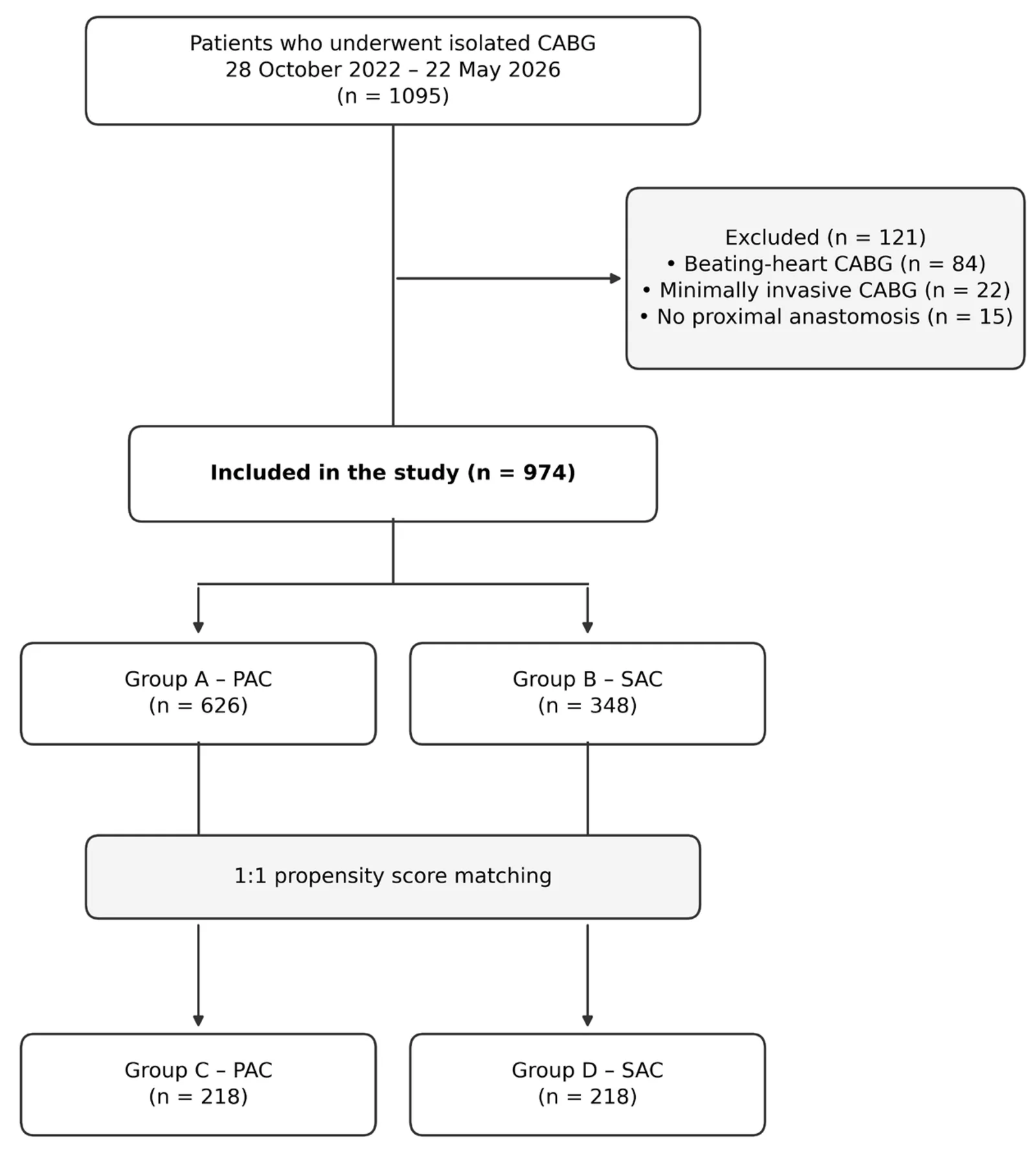
Flow diagram for study participants.

**Figure 2:**
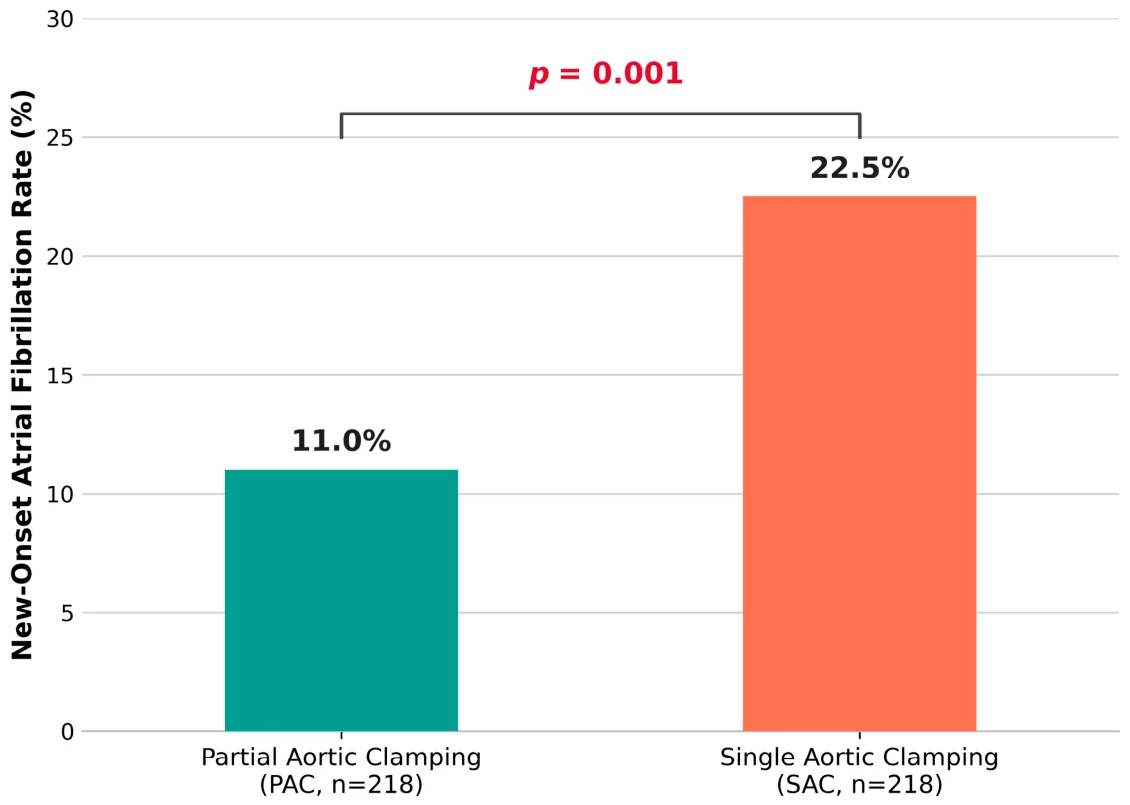
Comparison of new-onset atrial fibrillation incidence between partial aortic clamp and single aortic clamp techniques.

**Table 1:** Comparison of patient demographics, comorbidities, and operative data before and after propensity score matching.

	All Patients (n = 974)				*p*	Propensity Score-Matched (n = 436)				*p*	SMD
	Group A (n = 626) PAC		Group B (n = 348) SAC			Group C (n = 218) PAC		Group D (n = 218) SAC			
	Min–Max or n (%)	Median (IQR)	Min–Max or n (%)	Median (IQR)		Min–Max or n (%)	Median (IQR)	Min–Max or n (%)	Median (IQR)		
Demographic data											
Female sex	118 (18.8)		69 (19.8)		0.71	42 (19.3)		47 (21.6)		0.55	0.057
Age (years)	31–84	60 (13)	36–86	62 (13)	0.007	38–80	60 (12)	36–80	61 (13)	0.32	0.082
Height (cm)	137–196	170 (10)	135–185	170 (10)	0.34	142–185	169 (10)	147–185	170 (9)	0.58	0.081
Weight (kg)	50–126	80 (18)	45–127	80 (17)	0.33	54–123	80 (17)	53–118	80 (17)	0.75	0.012
Comorbid diseases											
Diabetes mellitus	353 (56.4)		196 (56.3)		0.98	126 (57.8)		127 (58.3)		0.92	0.009
Hypertension	375 (59.9)		212 (60.9)		0.75	137 (62.8)		133 (61)		0.69	0.038
COPD	38 (6.1)		27 (7.8)		0.31	11 (5)		16 (7.3)		0.32	0.095
Previous cerebrovascular accident	49 (7.8)		26 (7.5)		0.84	20 (9.2)		17 (7.8)		0.60	0.049
Chronic kidney disease	49 (7.8)		26 (7.5)		0.84	18 (8.3)		17 (7.8)		0.86	0.017
Preoperative atrial fibrillation	8 (1.3)		4 (1.1)		1.00	3 (1.4)		2 (0.9)		1.00	0.043
Carotid artery stenosis	49 (7.8)		31 (8.9)		0.55	16 (7.3)		18 (8.3)		0.72	0.034
Peripheral artery disease	74 (11.8)		47 (13.5)		0.44	26 (11.9)		28 (12.8)		0.77	0.028
Thyroid disease	45 (7.2)		22 (6.3)		0.60	11 (5)		19 (8.7)		0.13	0.145
Operation with DAPT	23 (3.7)		12 (3.4)		0.76	9 (4.1)		8 (3.7)		0.80	0.024
Preoperative ejection fraction (%)	20–65	60 (10)	25–65	55 (10)	0.92	20–65	55 (14)	25–65	55 (10)	0.99	0.004
Preoperative TAPSE (mm)	11–34	23 (4)	14–34	23 (4)	0.44	14–34	23 (4)	14–35	23 (4)	0.91	0.050
Postoperative ejection fraction (%)	15–66	55 (10)	20–60	55 (10)	0.99	25–65	55 (15)	25–60	55 (8)	0.67	–
Postoperative TAPSE (mm)	8–35	17 (5)	8–28	16.5 (5)	0.80	9–31	17 (5)	8–28	17 (4)	0.94	–
EuroSCORE II	0.6–31	1.3 (1.4)	0.6–42.4	1.5 (1.3)	0.25	0.6–19.2	1.5 (1.7)	0.6–24.9	1.5 (1.5)	0.96	0.031
Operative data											
Emergency surgery	43 (6.9)		23 (6.6)		0.87	18 (8.3)		11 (5)		0.17	0.129
Number of grafts	2–7	3 (1)	2–6	3 (2)	<0.001	2–5	3 (1)	2–6	3 (1)	0.89	0.016
LITA graft	569 (90.9)		303 (87.1)		0.06	195 (89.4)		193 (88.5)		0.76	0.029
XCL time (min)	22–202	67 (34)	28–217	101 (47)	<0.001	22–153	73 (38)	28–201	100 (49)	<0.001	–
CPB time (min)	40–479	122 (52)	51–287	136 (53)	<0.001	55–254	137 (56)	51–268	134 (52)	0.87	0.020
Before XCL NIRS right (%)	40–83	62 (9)	33–99	62 (10)	0.59	40–82	62 (10)	34–99	62 (12)	0.88	–
Before XCL NIRS left (%)	40–93	62 (8)	30–87	62 (11)	0.17	43–82	62 (10)	36–87	62 (12)	0.81	–
After XCL NIRS right (%)	34–83	59 (8)	32–82	59 (9)	0.48	45–76	58 (8)	32–79	58 (9)	0.89	–
After XCL NIRS left (%)	33–79	59 (8)	23–80	58 (11)	0.50	36–79	57 (8)	32–80	58 (11)	0.79	–

IQR: interquartile range, PAC: partial aortic clamping, SAC: single aortic clamping, COPD: chronic obstructive pulmonary disease, DAPT: dual antiplatelet therapy, TAPSE: tricuspid annular plane systolic excursion, EuroSCORE II: The European System for Cardiac Operative Risk Evaluation, LITA: left internal thoracic artery, XCL: cross-clamping, CPB: cardiopulmonary bypass, NIRS: near-infrared spectroscopy. SMD: standardized mean difference.

**Table 2:** Comparison of bleeding, and blood product between partial aortic clamp and single aortic clamp groups.

	PAC (n = 218)		SAC (n = 218)		*p*
	Min–Max or n (%)	Median (IQR)	Min–Max or n (%)	Median (IQR)	
Intraoperative red blood cells using	0–5	0 (2)	0–6	0 (2)	0.84
Intraoperative fresh frozen plasma using	0–4	0 (0)	0–4	0 (0)	0.40
Intraoperative platelet suspensions	0–2	0 (0)	0–2	0 (0)	0.08
Postoperative red blood cells using	0–15	0 (1)	0–12	0 (1)	0.46
Postoperative fresh frozen plasma using	0–7	0 (1)	0–9	0 (1)	0.007
Postoperative platelet suspensions	0–6	0 (0)	0–3	0 (0)	0.85
Postoperative amount of bleeding (mL)	150–3800	725 (400)	100–3250	700 (550)	0.09

**Table 3:** Comparison of laboratory parameters between partial aortic clamp and single aortic clamp groups.

	PAC (n = 218)		SAC (n = 218)		*p*
	Min–Max or n (%)	Median (IQR)	Min–Max or n (%)	Median (IQR)	
Preoperative laboratory parameters					
White blood cells (10^9^/L)	2.9–20	8.7 (3.1)	2–18.6	8.3 (3)	0.13
Hematocrit (%)	25–53	41 (7)	21–52	41 (6)	0.66
Platelets (10^9^/L)	64–939	248 (84)	63–586	250 (93)	0.86
Urea (mg/dL)	15–212	35.3 (18.3)	13.9–122.4	35.4 (13.8)	0.43
Creatinine (mg/dL)	0.46–6.2	0.92 (0.34)	0.27–9.98	0.93 (0.31)	0.20
Sodium (mEq/L)	128–152	139 (4)	125–145	139 (3)	0.49
Potassium (mEq/L)	3.4–5.8	4.3 (0.5)	3–6	4.2 (0.6)	0.24
Alanine aminotransferase (U/L)	3–222	19 (13)	1–196	17.5 (12)	0.07
Aspartate aminotransferase (U/L)	9–297	21 (14)	4–431	19 (10)	0.37
C-reactive protein (mg/dL)	0.2–246	4.4 (9.5)	0.3–192	4 (8.3)	0.81
HbA1c (mmol/mol)	4.9–16.2	6.4 (2.6)	4.7–14.3	6.3 (2)	0.79
Troponin T (ng/L)	3–1574	16 (58)	4–1864	18 (31)	0.55
CK-MB (IU/L)	0.5–139	1.6 (1.2)	0.4–72.2	1.6 (1.2)	0.73
Postoperative 1st day laboratory parameters					
White blood cells (10^9^/L)	3.7–44.2	16.2 (8.2)	5.3–50.3	16.1 (9.9)	0.44
Hematocrit (%)	20–40	28 (5)	20–60	28 (5)	0.88
Platelets (10^9^/L)	69–748	184 (69)	62–404	179 (77)	0.55
Urea (mg/dL)	18.2–149.2	40 (18.2)	13.3–108	41.5 (17.4)	0.45
Creatinine (mg/dL)	0.5–4.38	1.16 (0.43)	0.08–5.38	1.18 (0.47)	0.36
Sodium (mEq/L)	133–152	142 (4)	133–151	143 (5)	0.07
Potassium (mEq/L)	3–5.8	4.3 (0.7)	2.8–6.6	4.3 (0.7)	0.25
Alanine aminotransferase (U/L)	6–469	24.5 (20)	1–149	23 (29)	0.61
Aspartate aminotransferase (U/L)	16–602	56 (30)	16–559	54 (32)	0.46
C-reactive protein (mg/dL)	2.1–324	39.4 (25.6)	4.4–304	40.3 (27.7)	0.56
Postoperative 1st month laboratory parameters					
White blood cells (10^9^/L)	4.3–111	8.6 (3.5)	2.6–92	8.7 (3.1)	0.93
Hematocrit (%)	23–46	35 (6)	23–61	35 (6)	0.70
Platelets (10^9^/L)	57–775	373 (210)	31–817	354 (157)	0.92
Urea (mg/dL)	11–199	33.4 (16.7)	14.6–122.2	33.8 (15)	0.57
Creatinine (mg/dL)	0.5–5.86	1.16 (0.32)	0.4–7.17	1.18 (0.33)	0.83
Sodium (mEq/L)	125–150	139 (4)	129–145	138 (4)	0.96
Potassium (mEq/L)	3.2–5.9	4.5 (0.6)	3.4–6	4.6 (0.6)	0.22
Alanine aminotransferase (U/L)	3–353	17 (14)	4–5703	16 (11)	0.16
Aspartate aminotransferase (U/L)	7–353	16 (8)	7–7000	15 (8)	0.13
C-reactive protein (mg/dL)	0.7–181.4	19.1 (31.3)	1.2–383	16 (32.3)	0.90
Arterial blood gas					
Preoperative pH	7.31–7.52	7.41 (0.05)	7.27–7.55	7.41 (0.05)	0.79
Preoperative lactate (mmol/L)	0.4–35	1 (0.7)	0.3–3.8	1 (0.5)	0.93
Cross-clamp pH	7.28–7.56	7.41 (0.06)	7.28–7.51	7.40 (0.07)	0.29
Cross-clamp Hematocrit (%)	16–44	27 (6)	16–40	27 (6)	0.83
Cross-clamp potassium (mEq/L)	2.4–7.2	4.8 (1)	2.8–7.6	4.8 (0.9)	0.57
Cross-clamp lactate (mmol/L)	0.7–6.8	1.6 (1.1)	0.6–9.1	1.6 (1.2)	0.59
Postoperative day 1 pH	7.26–7.54	7.42 (0.05)	7.30–7.54	7.43 (0.05)	0.50
Postoperative day 1 lactate (mmol/L)	0.6–20	2 (1.5)	0.6–7.2	2 (1.6)	0.61

**Table 4:** Comparison of postoperative outcomes between partial aortic clamp and single aortic clamp groups.

	PAC (n = 218)		SAC (n = 218)		*p*
	Min–Max or n (%)	Median (IQR)	Min–Max or n (%)	Median (IQR)	
Postoperative exploration	14 (6.4)		12 (5.5)		0.68
Cerebrovascular accident	3 (1.4)		6 (2.8)		0.25
Continuous renal replacement therapy	7 (3.2)		4 (1.8)		0.36
NOAF	24 (11)		49 (22.5)		0.001
Percutaneous coronary intervention	2 (0.9)		4 (1.8)		0.34
Deep sternal wound infection	5 (2.3)		9 (4.1)		0.27
Gastrointestinal bleeding	1 (0.5)		1 (0.5)		1
IABP	5 (2.3)		3 (1.4)		0.36
ECMO	1 (0.5)		0		0.50
Tracheostomy	3 (1.4)		2 (0.9)		0.50
VIS	0–300	0 (7)	0–240	0 (7)	0.55
Intubation time (hours)	3–480	10 (7)	2–2184	10 (7)	0.67
Intensive care unit stay (days)	1–12	2 (1)	1–91	2 (1)	0.08
Hospital stay (days)	2–66	7 (3)	4–106	7 (3)	0.32
Mortality	5 (2.3)		7 (3.2)		0.55

IQR: interquartile range, PAC: partial aortic clamping, SAC: single aortic clamping, IABP: intra-aortic balloon pump, ECMO: extracorporeal membrane oxygenation, NOAF: new-onset atrial fibrillation, VIS: vasoactive inotropic score.

**Table 5:** Multivariate logistic regression analysis for new-onset atrial fibrillation.

	Odds Ratio	CI %95	*p*
Single aortic clamping	2.531	1.442–4.441	0.001
Age	1.092	1.055–1.130	<0.001
Female sex	0.871	0.431–1.759	0.70
Diabetes mellitus	0.557	0.307–1.010	0.054
Hypertension	1.372	0.745–2.526	0.31
Chronic obstructive pulmonary disease	0.744	0.253–2.187	0.59
Chronic renal failure	1.170	0.457–2.995	0.74
Preoperative ejection fraction	0.994	0.965–1.024	0.69
Emergency surgery	2.057	0.767–5.519	0.15
The number of grafts	0.979	0.661–1.450	0.92
Cardiopulmonary bypass time	0.999	0.991–1.007	0.80

CI, confidence interval.

## Data Availability

This database belongs to our clinic and is not publicly accessible.

## Supplementary Materials

The following supporting information can be downloaded at: https://www.mdpi.com/article/10.3390/jcdd13100502/s1, STROBE Statement—Checklist.

## Abbreviations

The following abbreviations are used in this manuscript:

CAD	Coronary artery disease
PCI	Percutaneous coronary intervention
CABG	Coronary artery bypass grafting
SAC	Single aortic clamp
PAC	Partial aortic clamp
XCL	Cross-clamp
CPB	Cardiopulmonary bypass
EF	Ejection fraction
TAPSE	Tricuspid annular plane systolic excursion
LITA	Left internal thoracic artery
RBCs	Red blood cells
FFP	Fresh frozen plasma
APS	Apheresis platelet suspension
CRP	C-reactive protein
CVA	Cerebrovascular accident
CRRT	Continuous renal replacement therapy
TTE	Transthoracic echocardiography
ABG	Arterial blood gas
NIRS	Near-infrared spectroscopy
ICU	Intensive care unit
NOAF	New-onset atrial fibrillation
DM	Diabetes mellitus
HT	Hypertension
AKI	Acute kidney injury
